# A Meta-Analysis of Odor Thresholds and Odor Identification in Autism Spectrum Disorders

**DOI:** 10.3389/fpsyg.2017.00679

**Published:** 2017-05-11

**Authors:** Maria Larsson, Carlos Tirado, Stefan Wiens

**Affiliations:** Gösta Ekman Laboratory, Department of Psychology, Stockholm UniversityStockholm, Sweden

**Keywords:** autism, olfactory sense, meta-analysis, odor threshold, odor identification

## Abstract

Autism Spectrum Disorders (ASD) are often accompanied by atypical visual, auditory, and tactile sensory behavior. Evidence also suggests alterations of the olfactory system, but the pattern of findings appears mixed. To quantify this pattern systematically, we conducted a meta-analysis. Studies were included if they examined olfactory function (i.e., odor threshold, or odor identification) in ASD compared with healthy age-matched control groups. We also coded for the potential moderators gender, age, and IQ. Articles were identified through computerized literature search using Web of Science, PubMed, and Scopus databases. A total of 11 articles compared odor threshold and/or odor identification between cases and controls (for threshold, *n* = 143 ASD and 148 controls; and for identification, *n* = 132 ASD and 139 controls). Effects sizes showed a substantial heterogeneity. As a result, the 95% prediction intervals were wide and ranged between a large negative and a large positive effect size for odor threshold, [-1.86, 2.05], and for odor identification, [-1.51, 2.52]. Exploratory analyses suggested that age and IQ may be potential moderators. To conclude, the large heterogeneity is consistent with the notion of both hyposensitivity and hypersensitivity in individuals with ASD. However, future research needs to predict and test the specific direction of the effect to provide convincing evidence for atypical olfactory functions in ASD.

## Introduction

Autism Spectrum Disorders (ASD) are characterized by social and communication difficulties, alongside repetitive behaviors and special interests (American Psychiatric Association, 2013). In addition, ASD is often accompanied by atypical sensory behavior (hyper- or hyporeactivity) for visual, tactile, and auditory information (Jones et al., 2003; Rogers et al., 2003). Given its high prevalence among individuals with ASD, unusual sensory processing was recently included in the DSM-5 diagnostic criteria for ASD (American Psychiatric Association, 2016).

Available evidence also suggests alterations of the olfactory system in ASD, but the pattern of findings is mixed. Inconsistent observations are reported for both sensory-driven olfactory tasks (e.g., odor threshold) and higher-order olfactory functions (e.g., odor identification). Although some studies reported either enhanced olfactory sensitivity in ASD (Ashwin et al., 2014) or impaired sensitivity (Dudova et al., 2011), most studies reported no significant differences between individuals with ASD and controls in olfactory threshold (Suzuki et al., 2003; Tavassoli and Baron-Cohen, 2012; Galle et al., 2013). Likewise, available evidence on odor identification is unclear, as some studies reported identification impairments (Suzuki et al., 2003; Wicker et al., 2016) whereas others reported no significant group differences (Dudova et al., 2011; Luisier et al., 2015).

Some degree of heterogeneity may be expected because previous studies used different methods, had small sample sizes, and may have sampled from different populations (e.g., age and gender). However, to determine if there is actual heterogeneity among the effect sizes that is not simply due to chance, we conducted a meta-analysis of existing studies examining olfactory function in individuals with ASD. We examined olfactory threshold and odor identification in ASD as compared with healthy age-matched control groups. Further, in an exploratory meta-regression, we investigated the potentially moderating role of available variables (i.e., gender, age, and IQ) upon the observed olfactory differences between ASD and controls.

## Materials and Methods

### Literature Search Strategy

Published studies were identified through computerized literature searches in Web of Science, PubMed, and Scopus databases for relevant studies targeting human olfaction in ASD, ASC (Autism Spectrum Condition), HFA (High-Functioning Autism), and AS (Asperger Syndrome). The search keywords were: autism and olfaction^∗^, ASD and olfaction^∗^, ASC and olfaction^∗^, HFA and olfaction^∗^, and AS and olfaction^∗^. The search was limited to English language articles. In addition, a manual review of articles was performed using cross-references from the original articles and reviews.

### Study Selection Criteria

Studies that were included in the meta-analysis focused on standard or experimental tasks of olfactory function in ASD, and had an age-matched control group of healthy participants without ASD (see **Figure 7**). Based on these criteria, two of the authors (ML and CT) and one research assistant independently reviewed and extracted data from each potential study. Disagreements were resolved via discussion of all authors until a consensus decision was reached. A total of 11 studies were included (see **Tables 1, 2**), and 15 studies were excluded (**Table 3**).

**Table 1:** Included olfactory threshold studies of Autism Spectrum Disorders

Author/year	Participants	Odor threshold test
Suzuki et al., 2003	ASD: *n* = 12; *M*_age_ = 32.9	*n*-Butanol;
	CG: *n* = 12; *M*_age_ = 30.8	custom-made
Dudova et al., 2011	ASD: *n* = 35 *M*_age_= 10.8	*n*-Butanol; SS-TT
	CG: *n* = 35; *M*_age_= 10.4	
Tavassoli and Baron-Cohen, 2012	ASD: n = 38 M_age_= 35.9	*n*-Butanol; SS-TT
	CG: n = 42; M_age_= 28.8	
Galle et al., 2013^∗^	ASD: *n* = 5; *M*_age_-= 21.4	*n*-Butanol;
	CG: *n* = 5; *M*_age_= 24.8	custom-made
Ashwin et al., 2014	ASD: *n* = 17; *M*_age_= 37.9	Alcohol Sniff Test
	CG: *n* = 17; *M*_age_= 27.2	
Kumazaki et al., 2016^∗^	ASD: *n* = 20; *M*_age_= 13.2	Isoamyl Acetate;
	CG: *n* = 23; *M*_age_= 12.5	Pulse-ejection
Addo et al., 2017	ASD: *n* = 16; *M*_age_= 38.2	*n*-Butanol;
	CG: *n* = 14; *M*_age_= 42.1	modified SS-TT

*The table lists only those studies on odor threshold (*k* = 7) included in the current meta-analysis. Studies are ordered by publication year. Participants [number of participants (*n*), mean age (*M*_*age*_)] and odor threshold test are included. ^∗^Results for one threshold test extracted. CG, control group; ASD, Autism Spectrum Disorder group; SS-TT, Sniffin’ Sticks-Odor Threshold Test; PEA, phenylethanol.*

**Table 2:** Included odor identification studies of Autism Spectrum Disorders.

Author/year	Participants	Odor identification test
Suzuki et al., 2003	ASD: *n* = 12; *M*_age_= 32.9	UPSIT (number of errors)
	CG: *n* = 12; *M*_age_= 30.8	
Bennetto et al., 2007	ASD: *n* = 21; *M*_age_= 14.3	SS-OIT
	CG: *n* = 27; *M*_age_= 14.5	
Brewer et al., 2008	ASD: *n* = 15; *M*_age_= 6.5	UPSIT
	CG: *n* = 15; *M*_age_= 7.1	
Dudova et al., 2011	CG: *n* = 35; *M*_age_= 10.4	SS-OIT
	ASD: *n* = 35; *M*_age_= 10.8	
Galle et al., 2013	ASD: *n* = 10; *M*_age_= 25.5	UPSIT
	CG: *n* = 11; *M*_age_= 22.0	
Luisier et al., 2015	ASD: *n* = 8; *M*_age_= 9.6	Custom-made
	CG: *n* = 10; *M*_age_= 10.0	
Wicker et al., 2016	ASD: *n* = 15; *M*_age_= 26.3	Custom-made
	CG: *n* = 15; *M*_age_= 27.8	
Addo et al., 2017	ASD: *n* = 16; *M*_age_ = 38.2	SS-OIT
	CG: *n* = 14; *M*_age_ = 42.1	

*The table lists only those studies on odor identification (*k* = 8) included in the current meta-analysis. Studies are ordered by publication year. Participants [number of participants (*n*), mean age (*M*_*age*_)] and odor identification test are included. CG, control group; ASD, Autism Spectrum Disorder group; UPSIT, University of Pennsylvania Smell Identification Test; SS-OIT, Sniffin’ Sticks-Odor Identification Test.*

**Table 3:** List of excluded studies.

Source	Reasons for exclusion
Rentowl and Hanning, 2004	Elderly only
Corcoran et al., 2005	Psychosis
Brewer et al., 2006	Book
Crane et al., 2009	General sensory processing
Lane et al., 2010	Subtypes of sensory processing
Silverman et al., 2010	Animal models
Brang and Ramachandran, 2010	Olfactory bulb and not autism
Hrdlicka et al., 2011	Hedonics
May et al., 2011	Reused data from Brewer et al. (2008)
Dudova and Hrdlicka, 2013	No control group
Martin and Daniel, 2014	Review
Rozenkrantz et al., 2015	Not relevant empirical data (sniffing)
Small and Pelphrey, 2015	Review
Liu et al., 2016	Nutrition in ASD
Nováková and Mrzílková, 2016	Healthy children

We targeted effect sizes for two basic olfactory domains: odor detection threshold and odor identification. For identification, most studies used the University of Pennsylvania Identification Test (UPSIT; Doty et al., 1984) or the Sniffin’ Sticks-Odor Identification Test (SS-OIT; Hummel et al., 1997). Both tests are standardized and widely used. In each task, each odor item is accompanied with four written label alternatives (one target and three foils), and the task is to pick the label that matches the presented odor. Of the selected studies, seven studies included a threshold test and eight studies used an identification test. Also, when available, we collected aggregated data on demographic (age and gender) variables and IQ.

### Statistical Analyses

Performance on the odor threshold task and on the odor identification task was compared between ASD and control groups. For each study, we extracted the relevant mean, standard deviation (or standard error), and sample size for each group. Because the studies used different measures, we computed the standardized, unbiased effect size estimate Hedges’ *g* (Lakens, 2013). If the necessary data were unavailable (for identification, *k* = 3), we used the reported inferential statistics to compute Hedges’ *g*. Hedges’ *g* and its estimated variance were computed (Lakens, 2013; Del Re, 2015) and then processed in the *R* toolbox *meta* (version 4.6-0) to perform the meta-analysis (Schwarzer, 2007; Schwarzer et al., 2015). Because study design and sampled population varied between the studies, a random-effects analysis was used to allow for heterogeneity of the true effect sizes among the studies, and this heterogeneity was estimated with DerSimonion–Laird estimator for *T*^2^ (Borenstein et al., 2009). The degree of heterogeneity was assessed with common measures such as *Q, I*^2^, and the prediction interval (Borenstein et al., 2009). We also performed exploratory meta-regressions in the *R* toolbox *meta* to investigate the potentially moderating role of several predictors. All data, *R* scripts, and results are available at Figshare (10.17045/sthlmuni.4801834) and OSF (10.17605/OSF.IO/V24NE), as recommended (Lakens et al., 2016).

## Results

**FIGURE 1:**
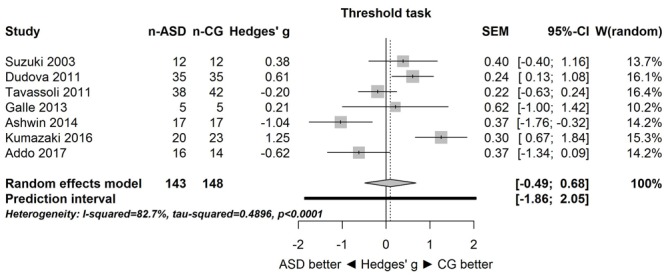
**Forest plot of performance on the odor threshold task in seven studies.** The prediction interval captures the expected true effect for a future study. For positive values, CG was better than ASD, and vice versa. ASD, autism spectrum disorder group; CG, control group. W(random) is the weight of each study in the random-effects model.

**FIGURE 2:**
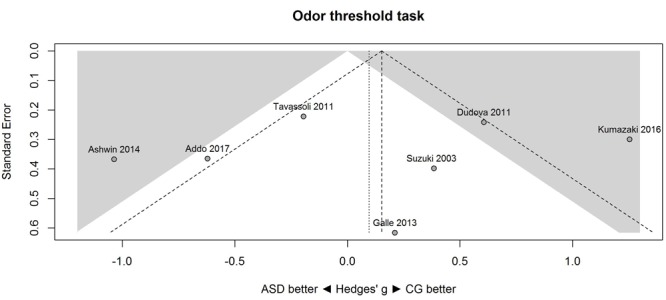
**Contour-enhanced funnel plot of performance on the odor threshold task in seven studies.** Studies that fall in the gray area are significant (at two-tailed alpha = 0.05). For positive values, CG was better than ASD, and vice versa. ASD, autism spectrum disorder group; CG, control group.

**Figure 1** shows the forest plot for the threshold task. Across studies, heterogeneity was substantial. Heterogeneity differed significantly from chance, *Q*(*df* = 6) = 34.73, *p* < 0.0001, *T* = 0.70, and about 83% of the observed variance reflected actual differences in the effect sizes, *I*^2^ = 82.7, 95% CI [65.7, 91.3]. The predicted effect size of a future study ranges between a large negative and a large positive effect size, as shown by the 95% prediction interval [-1.86, 2.05]. Consistent with this heterogeneity, the 95% CI of the mean estimate of the random-effects model was wide and overlapped zero, [-0.49, 0.68]. As shown in **Figure 2**, the heterogeneity is also illustrated in the contour-enhanced funnel plot (Schwarzer et al., 2015). Most studies had low precision and varied widely in either direction. The figure suggests that there was no particularly bias toward significant findings, which would be apparent if the studies fell mainly in the gray area (i.e., were significant at two-tailed alpha = 5%).

**FIGURE 3:**
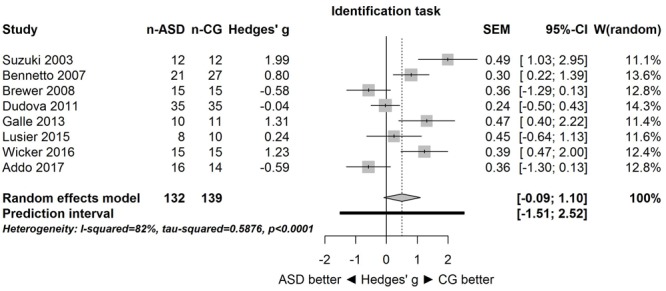
**Forest plot of performance on the odor identification task in eight studies.** The prediction interval captures the expected true effect for a future study. For positive values, CG was better than ASD, and vice versa. ASD, autism spectrum disorder group; CG, control group. W(random) is the weight of each study in the random-effects model.

**FIGURE 4:**
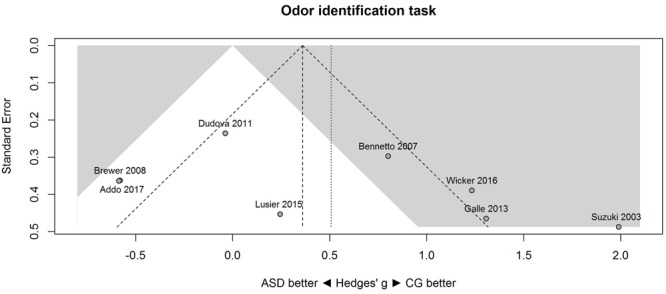
**Contour-enhanced funnel plot of performance on the odor identification task in eight studies.** Studies that fall in the gray area are significant (at two-tailed alpha = 0.05). For positive values, CG was better than ASD, and vice versa. ASD, autism spectrum disorder group; CG, control group.

A similar pattern of results was obtained for the identification task. **Figure 3** shows the forest plot for the identification task. Across studies, heterogeneity was substantial. Heterogeneity differed significantly from chance, *Q*(*df* = 7) = 38.87, *p* < 0.0001, *T* = 0.77, and about 82% of the observed variance reflected actual differences in the effect sizes, *I*^2^ = 81.9, 95% CI [65.7, 90.5]. The prediction interval ranged between a large negative and a large positive effect size, [-1.51, 2.52]. Consistent with this heterogeneity, the 95% CI of the mean estimate of the random-effects model was wide and overlapped zero, [-0.09, 1.10]. The heterogeneity is also illustrated in the contour-enhanced funnel plot (**Figure 4**). Most studies had low precision and varied widely. The figure suggests that there was no particularly bias toward significant findings.

Because for the identification task, one study (Suzuki et al., 2003) had a large effect size (Hedges’ *g* = 1.99), the above analysis was repeated without this study (i.e., sensitivity analysis). Results were unaffected: the large heterogeneity remained, *Q*(*df* = 6) = 26.94, *p* = 0.0001, *T* = 0.65, *I*^2^ = 77.7, 95% CI [53.7, 89.3], and 95% prediction interval, [-1.50, 2.14]. Consistent with this heterogeneity, the 95% CI of the mean estimate of the random-effects model was wide and overlapped zero, [-0.24, 0.87], although it was centered closer to zero than in the analysis of all studies.

Taken together, results showed that effect sizes varied substantially among the studies on both threshold and identification tasks. These results are consistent with the notion that ASD may lead to both hyposensitivity and hypersensitivity on these tasks. To identify potential moderators of the direction of this effect, we explored the contributions of three variables that we could extract from the studies in explaining the variability among effect sizes: proportion of males, mean age, and mean IQ score in the ASD group.

**FIGURE 5:**
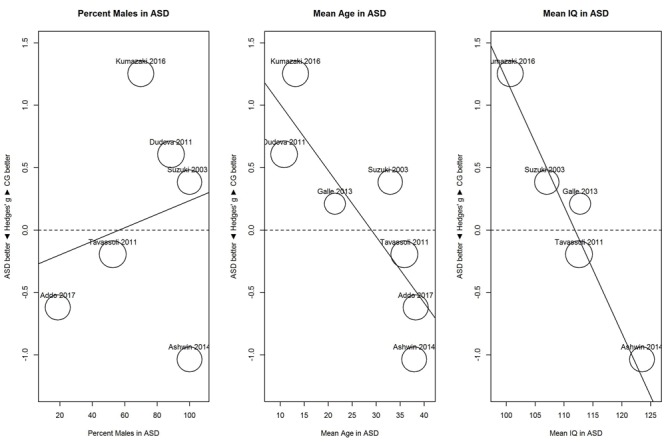
**Scatterplots of the relation with performance on the odor threshold task in terms of percent males in the ASD group (left panel), mean age in the ASD group (middle panel), and mean IQ in the ASD group (right panel).** For positive performance values, CG was better than ASD, and vice versa. ASD, autism spectrum disorder group; CG, control group.

Results of these exploratory meta-regressions showed that for threshold (**Figure 5**), proportion of males showed no clear relationship with sensitivity, *B* = 0.01, 95% CI [-0.02, 0.03], *R*^2^ < 0.01. In contrast, mean age and mean IQ tended to be associated with the performance difference between ASD and control group. For mean age, *B* = -0.05, 95% CI [-0.08, -0.02], *R*^2^ = 0.80; and for mean IQ, *B* = -0.10, 95% CI [-0.14, -0.06], *R*^2^ = 1.00. As shown in **Figure 5**, for younger participants (below 30), the ASD group (vs. the control group) showed hyposensitivity, whereas for older participants (above 35), the ASD group showed hypersensitivity. Similarly, for participants with IQs below 113, the ASD group showed hyposensitivity, whereas for participants with IQs above 113, the ASD group showed hypersensitivity. However, mean age and mean IQ correlated with each other (*r* = 0.72). Given the small number of studies, the unique contribution of each variable cannot be estimated.

**FIGURE 6:**
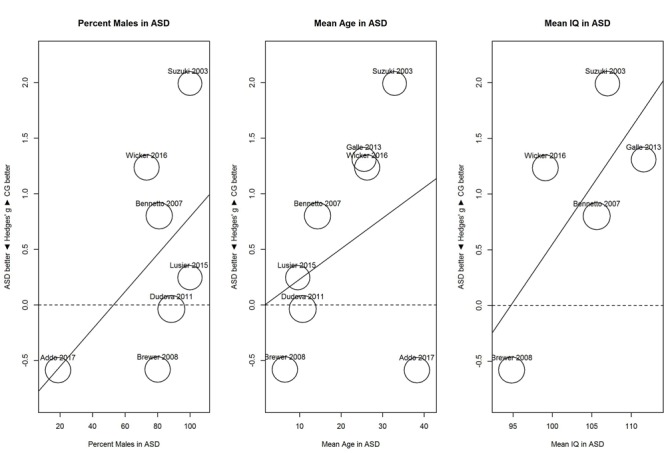
**Scatterplots of the relation with performance on the odor identification task in terms of percent males in the ASD group (left panel), mean age in the ASD group (middle panel), and mean IQ in the ASD group (right panel).** For positive performance values, CG was better than ASD, and vice versa. ASD, autism spectrum disorder group; CG, control group.

For identification (**Figure 6**), proportion of males and mean age showed no clear relationship with sensitivity; for proportion of males, *B* = 0.02, 95% CI [-0.01, 0.04], *R*^2^ = 0.03; and for mean age, *B* = 0.03, 95% CI [-0.03, 0.08], *R*^2^ < 0.01. In contrast, mean IQ tended to be associated with the performance difference between ASD and control group; for mean IQ, *B* = 0.10, 95% CI [-0.01, 0.21], *R*^2^ = 0.45. As shown in **Figure 6**, with increases in mean IQ, the ASD group increased in hyposensitivity.

**FIGURE 7:**
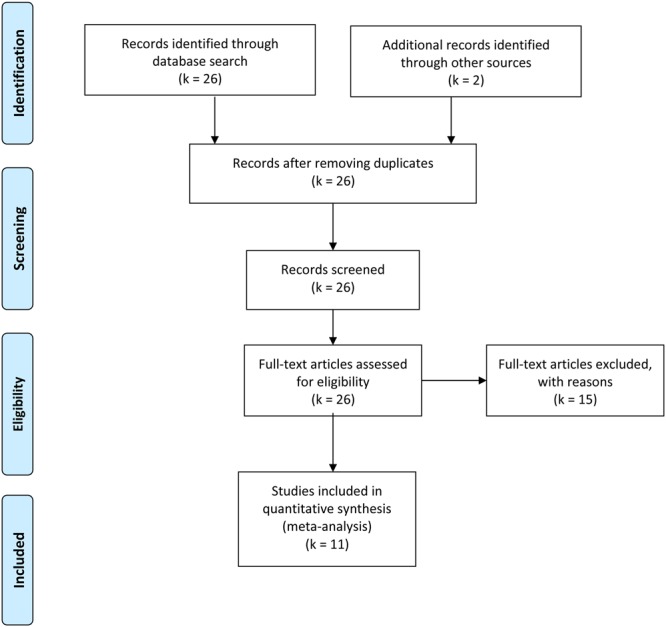
**Publications’ selection process**.

## Discussion

The goal of the present study was to summarize quantitatively the empirical evidence for changes in olfactory function in individuals with ASD. Although only few studies with small sample sizes were available, the meta-analysis provided strong evidence that the effect sizes varied substantially among the studies for both threshold and identification tasks. This heterogeneity is clearly apparent in the large 95% prediction intervals (see **Figures 1, 3**) for the threshold task [-1.86, 2.05] and for the identification task [-1.51, 2.52]. These intervals imply that for a future study, the true effect size may fall between very negative and very positive. As such, the results are consistent with the idea of both hyposensitivity and hypersensitivity in individuals with ASD.

To investigate this heterogeneity, we explored the role of potential moderators that may be associated with hyposensitivity and hypersensitivity: proportion of males, mean age, and mean IQ score in the ASD group. For threshold, mean age below 30 and mean IQ below 113 tended to be associated with hyposensitivity whereas mean age above 35 and mean IQ above 113 tended to be associated with hypersensitivity. However, mean age and mean IQ correlated with each other (*r* = 0.72). This makes it difficult to separate the unique contribution of each variable. For identification, increases in mean IQ were associated with increased hyposensitivity. These results are surprising because they suggest that mean IQ is associated with opposite effects depending on the task: increased mean IQ is associated with hypersensitivity on a threshold task and hyposensitivity on an identification task. However, these findings from the meta-regression need to be interpreted carefully (Borenstein et al., 2009). First, they are based on few studies with low precision and thus, the findings are very tentative. Second, they are based on mean differences in the groups and may not apply to individual data (i.e., ecological fallacy). Third, mean IQ may be only a correlate of the underlying, yet unknown mechanism.

Given the substantial heterogeneity among observed effect sizes, future research should focus on a particular group of individuals to establish hyposensitivity or hypersensitivity (or neither). Notably, the present results imply that 35-year old males with ASD and an IQ around 115 would show opposite effects on threshold and identification tasks, that is, hypersensitivity on the threshold task and hyposensitivity on the identification task. To determine if this is not simply a chance finding, future research should study well-characterized individuals with standardized methods and consider the role of potential moderators. Any potential moderators should be reported completely to avoid missing data. Sample size should be large to reduce the uncertainty in the obtained estimate. Although a power analysis is often recommended to determine sample size, a power analysis seems cumbersome in the present context, as the effect sizes were heterogeneous and their direction was unclear. Critically, a power analysis is meaningful only from the perspective of null hypothesis significance testing with its focus on the *p*-value. In contrast, a Bayesian approach would allow researchers to analyze the data after every participant (Wagenmakers et al., 2015). This simplifies data collection because participants would not have to be recruited unnecessarily. In the Bayesian approach, participants might be recruited until the evidence is strong (e.g., Bayes Factor > 10). In the analysis, a default *prior* could be used that predicts only the direction of the effect (hyposensitivity or hypersensitivity), and subsequent analyses could evaluate the robustness of the findings depending on the prior (Wagenmakers et al., 2016). Another advantage is that Bayesian analysis can provide support for the null hypothesis (i.e., there is no group difference), something that null hypothesis significance testing cannot provide, even in high-powered studies (Dienes, 2016); for an example in addiction research, see here (Beard et al., 2016). Also, Bayesian analyses permit the calculation of credible intervals (Wiens and Nilsson, 2016). These capture the actual precision of the estimate, whereas it is incorrect to interpret confidence intervals in terms of precision (Morey et al., 2015). Furthermore, the study should be pre-registered and designed to minimize risk of bias (Nosek and Lakens, 2014; Nuzzo, 2015). Last, the raw data should be available online to facilitate future meta-analyses (Nosek et al., 2015; Munafò et al., 2017). In particular, meta-analyses that use the raw data (rather than aggregated data) can model individual data to avoid typical fallacies such as the ecological fallacy (Lakens et al., 2016).

## Conclusion

The present meta-analysis provides convincing evidence for heterogeneity among effect sizes that exceeds that expected by chance. Demographic variables such as age and IQ may account, at least in part, for this heterogeneity, and their effects may differ depending on the task. Future studies should be conducted on well-defined samples with standard methods to assess olfactory functions in ASD. If a study is pre-registered and uses Bayesian statistics, participants could be added consecutively until the evidence is convincing for hyposensitivity, hypersensitivity, or neither.

## Author Contributions

ML and SW developed the study concept. ML and CT jointly searched the databases and extracted the data. SW analyzed the data and produced the figures. ML, CT, and SW wrote the manuscript.

## Conflict of Interest Statement

The authors declare that the research was conducted in the absence of any commercial or financial relationships that could be construed as a potential conflict of interest.
